# Facial Anthropomorphic Trustworthiness Scale for Social Robots: A Hybrid Approach

**DOI:** 10.3390/biomimetics8040335

**Published:** 2023-07-29

**Authors:** Yao Song, Ameersing Luximon, Yan Luximon

**Affiliations:** 1 Digital Convergence Laboratory of Chinese Cultural Inheritance and Global Communication, Sichuan University, Chengdu 610065, China; yao.song@scu.edu.cn; 2 College of Literature and Journalism, Sichuan University, Chengdu 610065, China; 3 School of Design, The Hong Kong Polytechnic University, Hung Hom, Hong Kong 999077, China; 4 Georgia Tech Shenzhen Institute, Tianjin University, Shenzhen 518071, China; luximon@gtsi.edu.cn

**Keywords:** artificial intelligence, social robot, face, scale

## Abstract

Social robots serve as autonomous systems for performing social behaviors and assuming social roles. However, there is a lack of research focusing on the specific measurement of facial trustworthiness toward anthropomorphic robots, particularly during initial interactions. To address this research gap, a hybrid deep convolution approach was employed in this study, involving a crowdsourcing platform for data collection and deep convolution and factor analysis for data processing. The goal was to develop a scale, called Facial Anthropomorphic Trustworthiness towards Social Robots (FATSR-17), to measure the trustworthiness of a robot’s facial appearance. The final measurement scale comprised four dimensions, “ethics concern”, “capability”, “positive affect”, and “anthropomorphism”, consisting of 17 items. An iterative examination and a refinement process were conducted to ensure the scale’s reliability and validity. The study contributes to the field of robot design by providing designers with a structured toolkit to create robots that appear trustworthy to users.

## 1. Introduction

As an intermediary communication tool between artificial intelligence (AI) and humans, a social robot serves as an autonomous system capable of performing social behaviors or assuming various social roles [1]. In contrast to industrial robots, which often possess limited human-like characteristics, modern social robots are typically physically embodied with a head and associated facial features [2,3,4,5]. By utilizing specific motors and sensors, social robots can fulfill social roles such as assisting humans with specific tasks, responding to people’s needs, and even providing emotional companionship and support [6]. Trustworthiness evaluation, an essential personality attribute, involves assessing the honesty and reliability of an individual or object [7]. Just as trustworthiness is crucial in interpersonal interactions, it also plays a significant role in human–robot interaction (HRI) because social robots not only function as passive “helpers” capable of physical assistance but also act as active “caregivers” capable of emotional support for individuals [8].

Furthermore, humans have a strong inclination to detect human faces or anthropomorphic features [9]. However, there is limited research exploring the specific measurement of facial trustworthiness toward anthropomorphic robots, particularly upon initial encounters. Considering the growing number of social robots designed to provide emotional support and companionship to humans [10], it is reasonable to assume that similar affective responses and personality evaluations may arise in relation to these robots [11]. Unfortunately, this aspect has been largely overlooked in current robot design and research studies. Therefore, this study aims to address this research gap by employing a hybrid approach to develop a scale for measuring the facial anthropomorphic trustworthiness of social robots.

## 2. Literature Review

### 2.1. Facial Anthropomorphic Trustworthiness

The question of “Should I trust this person?” often arises immediately after encountering a stranger, particularly when we heavily rely on first impressions. This process is known as trustworthiness at first sight, which primarily involves an impulsive evaluation of trustworthiness based on specific facial features [12]. Research suggests that people can make trustworthiness evaluations and subsequent judgments solely based on facial appearance [13]. Surprisingly, individuals also tend to be influenced by facial appearance even in situations where important decisions need to be made, such as voting for a political leader. During these events, people are expected to gather diverse information, utilize their cognitive abilities, and make rational choices [12]. However, in reality, when faced with cognitively complex issues and limited or overwhelming information, individuals often prefer to simplify the decision-making process by relying on easily processed attributes, thereby reducing effort and cognitive strain associated with social judgments [14].

Naturally, humans have a tendency to detect and recognize faces [15]. This inclination, however, extends beyond human faces and includes human-like objects such as social robots [16]. A recent eye-tracking study compared gaze fixation patterns when evaluating the faces of a real human and a robot, suggesting that there were no significant differences in gaze fixation between humans and robots [17]. Social robots, unlike industrial machines or robots, are often designed to have humanoid shapes and features, which are believed to bear a closer resemblance to humans [18]. For example, prior research has shown, consistent with human cognition, that robots with specific facial features, expressions, and social cues evoke more trust [19,20,21]. Consequently, humans are more likely to attribute positive and favorable personality traits, such as trustworthiness, to these robots [22].

Taking a closer look at the perceived trustworthiness of these emerging media, social robots can be seen as a medium for the self-completion of humans [23]. Interpreting an object can offer us a different perspective of ourselves, going beyond the object itself [24]. In fact, it represents a shift from a history-based viewpoint to a human-oriented viewpoint [23]. As stakeholders in the process of trustworthiness evaluation, individuals can shape this evaluation process and, in turn, be influenced by it, prompting a reconsideration of the boundary between themselves and the object [25]. Recent neuroscientific research provides further evidence supporting this view, suggesting that individuals may consider their possessions as representations of their identities [26]. Accordingly, embracing an object that is consistent with one’s identity can help build an ideal self-image, not only for personal satisfaction but also to maintain a consistent impression in the public eye. This may explain the preference for social robots with a trustworthy appearance.

### 2.2. Trustworthiness Dimensions for Social Robot

It is natural to assume that facial anthropomorphic trustworthiness shares some common dimensions with interpersonal trustworthiness [27]. Mayer and his colleagues [28] proposed three dimensions of interpersonal trustworthiness: ability, benevolence, and integrity. These dimensions are conceptually distinct from each other as they encompass both cognitive and affective attributes of trustworthiness [29]. While these dimensions provide a theoretical framework for understanding trustworthiness, they may be somewhat limited in scope, especially in the context of social robot research, where additional dimensions could be relevant and complementary.

Specifically, ability in interpersonal trustworthiness refers to an individual’s evaluation of others’ competence and knowledge in a given task [28]. This evaluation represents the initial expectation of others’ expertise or endorsement (e.g., trusting a doctor based on their academic credentials). In the context of human–robot interaction (HRI), it may pertain to the belief that a social robot has the necessary capabilities to fulfill its intended functions [30].

Integrity, on the other hand, refers to an individual’s assessment of whether others will adhere to a set of social rules in interpersonal interactions [28]. This evaluation involves the initial confidence in others’ behavior and perceived risk. In the context of HRI, people may have ethical concerns regarding robots. On one hand, they may be concerned about the integrity of the robot’s creator or designer, as they are considered “the man behind the curtain” [31]. On the other hand, individuals may express anxiety about the self-awareness of such emerging technologies and even harbor doubts about their ability to adhere to programmed rules [32].

Benevolence, the third dimension, relates to the extent to which individuals are inclined to act kindly toward others, even beyond self-interest [28]. This evaluation reflects affective expectations regarding the kindness and goodwill of others in physical and psychological interactions. In the context of HRI, people may expect social robots to possess altruistic and emotionally approachable personalities [33].

In addition to the dimensions of facial anthropomorphic trustworthiness in social robots, there are unique characteristics that are significant for robot design, such as the “uncanny valley” effect [34,35]. This phenomenon describes the non-linear relationship between people’s emotional reactions to robots and the degree of resemblance to humans. People often feel unsettled when encountering robots with imperfect human-like features, especially in facial expressions [36]. Consequently, it is plausible that individuals might prefer and trust a robot with appropriately designed facial features that neither appear too robotic nor too humanoid. However, empirical evidence supporting this notion is somewhat lacking and requires further investigation.

## 3. Methods

**Figure 1:**
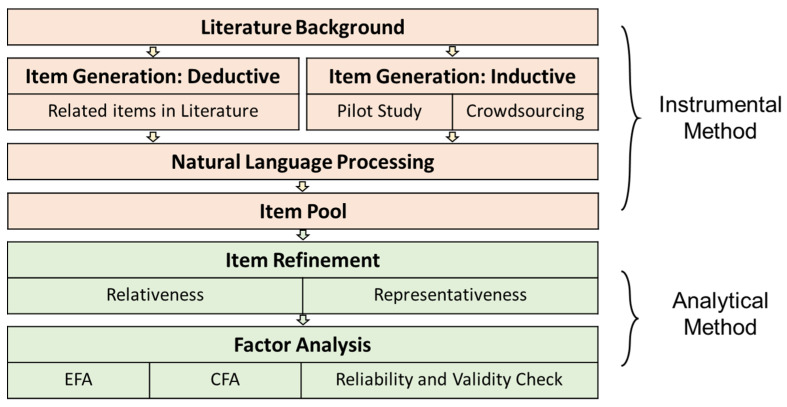
Method overview of this study.

To develop the scale of facial anthropomorphic trustworthiness for social robots, instrumental methods (content analysis, crowdsourcing collection, and NLP) and analytical methods (exploratory factor analysis, confirmatory factor analysis) were used to address our research aim. Specifically, based on the literature review, we first ran a pilot study (interview) to provide preliminary evidence of various constructs of facial anthropomorphic trustworthiness and further confirmed the validity of the questions used in the second step. Then, we distributed the questionnaire via a crowdsourcing platform to collect a relatively large sample of qualitative data. With the help of the state-of-art (SOTA) natural language processing technique, the qualitative data were clustered into several clusters. Together with the items retrieved from the literature, we formed the initial item pool. Last, we followed the standard scale development procedure to form the item pool and further examined and validated the validity of our scale via EFA, CFA, etc. (Figure 1).

### 3.1. Instrumental Methods

#### 3.1.1. Traditional Methods vs. Crowdsourcing to Explore User Experience

Before launching a new product or service, user experience teams would like to utilize traditional qualitative methods, such as in-depth interviews or focus groups, to explore insights into user experience that reflect their opinions toward the given product or service [37]. Based on the collected data, trained researchers would screen the transcript, remove the redundancy, and form the initial corpus for further review. Through manual coding and clustering, the statements of the corpus would be grouped into certain themes. Sometimes, the generated themes even could have a hierarchical relationship [38]. Relying on the extracted themes, researchers could identify and summarize user experience.

Although the traditional method has enjoyed sufficient reliability and validity in exploring user experience, it still might face some problems: a relatively small sample [39], time-consuming, and expensive [40]. In order to address this problem, the latest qualitative research tries to utilize various data sources to collect qualitative data, such as user-generated content (UGC) and crowdsourcing platforms. Although user-generated content (UGC) is a data collection source, its quality varies to a large extent and it mainly depends on a specific website and cannot reach the particular population out of the given theme on the website [41]. Different from UGC, a crowdsourcing platform could actively collect qualitative data, which might be of a similar quality to the traditional method in various disciplines [42]. Specifically, crowdsourcing, a combined word of “crowd” and “outsourcing”, refers to distributing microtasks to others online [43]. Based on personalized service from the workers, crowdsourcing is very helpful since it could provide diverse, innovative, and numerous solutions [44].

Among all the crowdsourcing platforms, Amazon Mechanical Turk is the largest and the most popular one, which has more than 800,000 registered workers (commonly called “Mturkers”) across over 200 countries with more than 600,000 microtasks (so-called “Human Intelligence Tasks”, or “HITs” for short) per day [45]. Considering the number, age, diversified locations, and affiliations of registered Mturkers, Mturk has become an efficient tool and a reliable data collection source for scientific research, ranging from recruiting behavioral experiment participants [46], labeling specific images or videos [47], and retrieving consumer insight [48]. Much research has suggested that its data show adequate quality and reliability even compared with physical lab experiments [44].

#### 3.1.2. Natural Language Processing (NLP) in Deep Convolution

**Figure 2:**
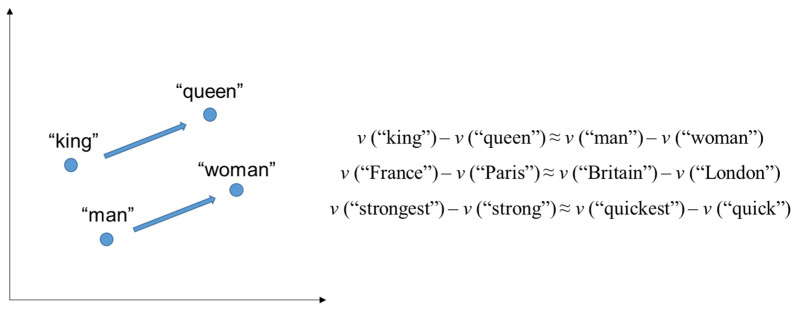
Word representation example in vector space.

Natural language processing (NLP) is a multidisciplinary field of artificial intelligence, languages, information extracting and representation, and data science, which aims to explore the associations between mathematical representations and natural languages, especially in the field of processing and analyzing a large-scale human language via coding [49]. There are generally two subfields of NLP techniques that are related to this study: word2vec (word-to-vector) embedding and bidirectional encoder representations from transformers (BERT). Semantic words or sentences are trained to be mathematically represented (vectorized) as real-valued mappings (around 20–400 dimensions). In this way, similar words or sentences are mathematically close to each other in the vector space [50]. This technique indicates the mechanism in the word2vec or sentence embedding (either average, sum, or contacting a set of word vectors to produce sentence embedding): words or sentences which co-occurred in a similar context would share similar linguistic connotations [50]. Moreover, after training a large number of a specific corpus, word2vec embedding could reveal not only the extent of similarity between words or sentences but also the semantic relationships between words and sentences [51]. For example, after training on the corpus of Google News, word2vec embedding could reflect the following relationship: the vector distance between the word “king” and “queen” is similar to the vector distance between the word “man” and “woman” [51] (Figure 2).

However, traditional word2vec has two main drawbacks: it cannot solve either word polysemy problems or complex characteristics of a sentence. Word2vec starts from the distributed hypothesis of word meaning (the meaning of a word is given by words that frequently appear in its context), and the result is a look-up table, where each word is associated with a unique dense vector [50]. Indeed, each word in different contexts may have different meanings: its numerical values should not be a fixed vector [52]. However, the word representation generated by word2vec is static, regardless of context. In other words, a look-up-styled word2vec embedding is difficult to adapt and perform well to all downstream tasks; thus, a variety of adapted models are introduced for different tasks, which are basically generated by adding their own inductive biases for each task [53].

In order to address those drawbacks of word2vec, in 2018, Jacob Devlin and the research team [54] from Google Co. introduced BERT as a state-of-art (SOTA) technique for NLP. Generally speaking, BERT is a method of pre-training language representations, namely, a general “language comprehension” model trained through numerous corpora, such as Wikipedia (around 2500 million words) and a book corpus (around 800 million words), and then is utilized for downstream language tasks (after fine-tuning), such as classification and autonomous conversions [54]. The major innovation of BERT is the proposed pre-train method. To specify, it relied on mask language modeling (MLM) and next sentence prediction (NSP) to capture the representation of text and sentence level, respectively. Compared with traditional word2vec embedding, BERT has two significant advantages: on one hand, BERT uses a transformer (encoder) as a feature extractor to solve the polysemy problem by representing each word as a function of the whole sentence and extracting context information for each word in the forward and backward directions [55]. Cooperating with de-noising targets such as a masked language model (MLM) on large-scale corpora, the generated representations are constructive for downstream tasks, such as classification. Therefore, compared with the word embedding method represented by word2vec, BERT has a more noticeable improvement which is more dynamic and can model the phenomenon of polysemy. On the other hand, pre-trained models are designed to include different levels of language features at different network layers since different tasks rely on different levels of features differently [54]: some tasks might rely on more abstract information, while others focus more on grammatical information. In this way, BERT can selectively use the information at all levels, which could reflect different levels of features on different network layers due to its learning in a “deep” network [56].

### 3.2. Analytical Methods

Exploratory factor analysis (EFA) and confirmatory factor analysis (CFA) were used as analytical methods to explore the nature of facial anthropomorphic trustworthiness. To specify, EFA is a statistical technique used to explore the underlying structure of a set of observed variables or items and identify the latent factors that account for their covariation [57]. EFA is commonly employed in scale development to assess the construct validity of a measure by determining the number of factors or dimensions underlying a set of items [58]. Through this method, researchers can uncover the relationships between items and group them into factors that represent different aspects of the construct being measured. Before conducting EFA, researchers typically assess the suitability of their data through two preliminary tests: the Kaiser–Meyer–Olkin (KMO) measure of sampling adequacy and Bartlett’s test of sphericity. As for CFA, it is a statistical technique used to evaluate the fit between a hypothesized factor structure and the observed data [59]. Unlike EFA, which is exploratory in nature, CFA is used to confirm or validate a pre-specified factor structure derived from theory or previous research. In scale development, CFA is utilized to test the construct validity of a measure by assessing whether the observed data support the proposed factor structure and the relationships between the items and their respective factors [60].

In summary, EFA, along with the preliminary tests of KMO and Bartlett’s test, allows researchers to explore and determine the underlying factor structure of a set of items. CFA, on the other hand, verifies the adequacy of the hypothesized factor structure.

## 4. Different Phases in Developing and Validating Scale

### 4.1. Phase 1: Item Generation via a Hybrid Method

**Figure A1:**
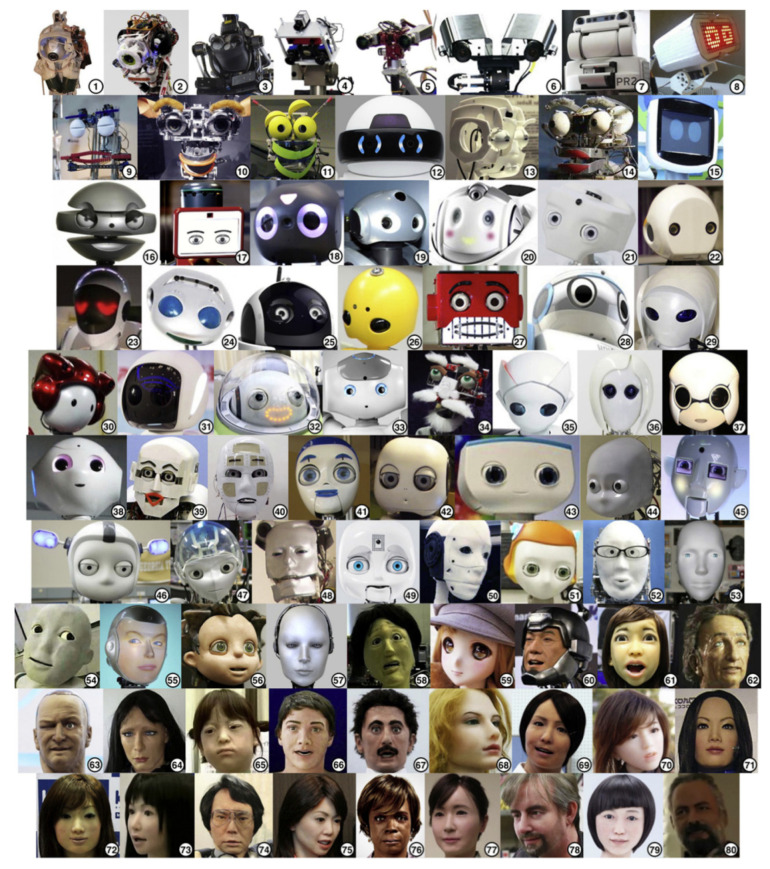
The 80 Robot Facial Stimuli in This Study [22].

Before the main study, we initially conducted a pilot study via a convenience sample of ten participants (mean age = 31.5; 5 Chinese and 5 non-Chinese; 2 males and 8 females). Participants were first informed about the aim and were instructed to view a set of 80 robot faces [22] (see Figure A1). To be more specific, the facial stimuli were preloaded onto tablets, and participants were instructed to pay attention to them for a specified duration, typically 10 min. Then, the interviewer explored their experience with the set of robot faces and the reasons a robot looked trustworthy or untrustworthy. After fully transcribing and manually coding, the content analysis showed that (1) participants agreed that they have an unconscious facial trustworthiness evaluation of social robots at first sight, and (2) four themes (“capability”, “positive affect”, “ethics concern”, and “anthropomorphism”) emerged and were partially consistent with previous literature [28]. Aligned with prior research, these four distinct dimensions are significant constructs to measure trustworthiness in HRI. Detailed definitions and sources can be found in Table 1. Thus, a deep-learning-based theme generation was adopted to a larger sample to confirm the finding in the pilot study.

For the main study, the scale development process started by generating an item pool for further exploration. This process could be conducted by two approaches: a deductive approach (e.g., retrieving items from previous literature) and an inductive approach (e.g., retrieving items from an in-depth interview or focus group). With a deductive approach, two researchers familiar with the literature on HRI, human cognition and perception, and psychology theory conducted an extensive literature review on facial anthropomorphic trustworthiness. They discussed, theorized, and summarized all the related items from prior studies, then collected and removed the replicated items, and finally agreed on four main themes which are consistent with the findings in the pilot study. With the inductive approach, a questionnaire based on the pilot study was distributed via AMT, and 200 participants (mean age = 36.31, SD = 10.47; 112 males and 88 females; education level: 19 with high school education or lower, 60 with some college, 122 with college education or above; robot use experience: 128 with no use, 39 with 0–1 year of use (1 year not included), 19 with 1–2 years of use (2 years not included), 14 with more than 2 years of use) were recruited to give their opinions on facial anthropomorphic trustworthiness. Specifically, they initially informed the general information of this study. Participants in this research were asked to report their demographic information and prior robot interaction experience. Lastly, they were exposed to a set of robot faces from the previous database [22] and were asked the same questions in the pilot study.

**Figure 3:**
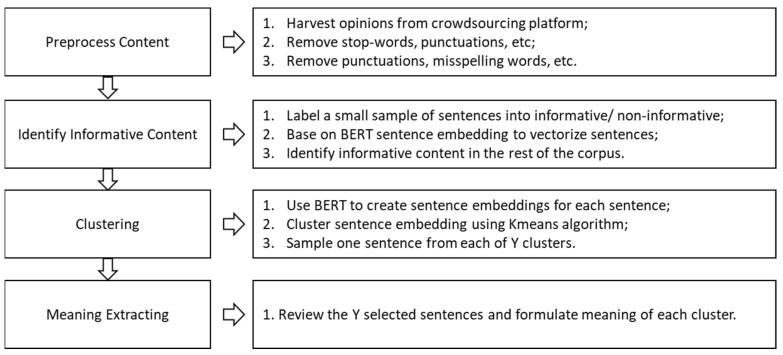
The architecture of the item pooling process.

Next, we utilized NLP techniques to process the collected responses in four steps: preprocessing the content, identifying informative content, clustering, and meaning extracting (Figure 3).

(1) Preprocess Content.

Previous qualitative research has suggested that each sentence in the corpus is a natural unit that could potentially reflect user opinion or experience [61]. Thus, all the qualitative responses from AMT were split into a set of sentences via an unsupervised sentence split toolkit [62]. Then, we cleaned them by removing stop-words; converting all letters to lowercase; transferring numbers into number signs; removing punctuation, accent marks, and other diacritics; removing white spaces; and processing abbreviations [63].

(2) Identify Informative Content.

In this stage, we labeled a relatively small set of sentences into two categories (informative/non-informative) and then applied BERT embedding to each sentence, filtering out non-informative sentences from the rest of the corpus. With the BERT identification, researchers would focus more on informative sentences [63].

(3) BERT sentence embedding clustering.

Regarding the clustering tasks, a commonly used method is to tokenize each sentence to a vector space in which semantically similar sentences have a closer distance to each other. Previous research has tried to put sentences into BERT and retrieve the fixed-sized BERT embedding. For example, Reimers and Gurevych [64] fine-tuned BERT with a Siamese and triplet structure to develop more semantically meaningful BERT embedding which could be distinguished by its cosine-similarity. Because Siamese BERT-Networks have shown efficacy in computing sentence similarity, we also used the same structure of BERT in this application [64].

Considering similar sentences should have a close distance in the BERT embedding vector space, the set of sentences was then grouped into the cluster via the K-means clustering algorithm, which is a method of vector quantization commonly used in text-clustering studies [65]. To specify, K-means clustering is to divide n observations (*x*_1_, *x*_2_, …, *x_n_*) into *k* (≤*n*) set *S* = {*S*_1_, *S*_2_, …, *S_k_*}, ensuring the minimization of the within-cluster sum of squares.

$$argmin\sum_{i=1}^{k}\sum_{x\in S_{i}}{\left\|x-n_{i}\right\|}^{2}=argmin\sum_{i=1}^{k}\left|S_{i}\right|VarS_{i}$$

where *ui* is the mean of points in set *S_i_*.

**Figure 4:**
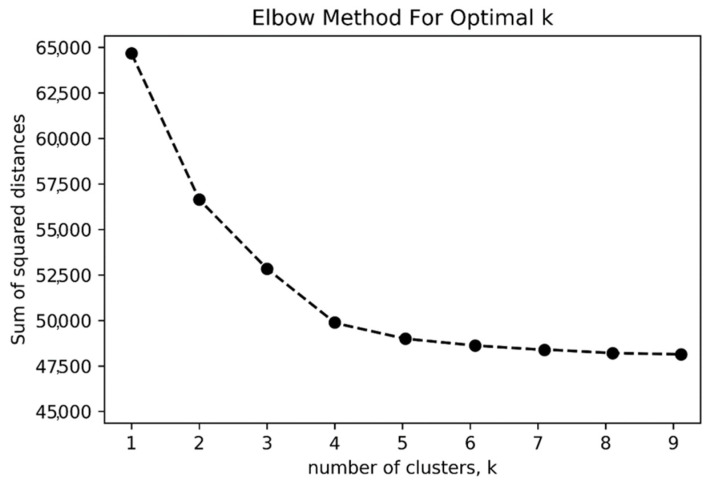
The elbow method to determine the optimal number of clusters.

To identify an optimal number of clusters, the elbow method has been widely used to determine Y clusters [66]. This method relies on calculating the sum of squared distance as different clusters of *k* increase to choose the optimal number of *k* when the sum of squared distance is only reduced marginally. As shown in Figure 4, four might be an appropriate number of clusters for this dataset, which is also consistent with the results in the pilot study and inductive approach [67].

**Table 1:** The source and example of the item pool.

Variables	Definition	Methods	Source and Example
**Ethics** **Concern**	Ethics concern refers to the extent to which individuals perceive that the robot has been designed and programmed with ethical considerations in mind. It involves the evaluation of whether the robot’s actions, behaviors, and decision-making processes align with ethical principles or values.	Inductive	Schaefer [68], Hancock et al. [69], Tay et al. [70], Wheless and Grotz [71], Colquitt and LePine [72], Yogoda and Gillan [73], Bhattacherjee [39], Büttner and Göritz [74]
		Deductive	*“I’d trust the one learned from a compassionate creator in a safe loving environment”* *“People can write various codes and programs to make robots do evil things”*
**Capability**	Capability refers to the dimension that assesses individuals’ perceptions of a social robot’s competence and ability to perform its designated tasks or functions effectively.	Inductive	Schaefer [68], Hancock et al. [69], Tay et al. [70], Wheless and Grotz [71], Colquitt and LePine [72], Yogoda and Gillan [73], Bhattacherjee [39], Büttner and Göritz [74]
		Deductive	*“They are good robots and competent enough to their programmed task”* *“I want to see them as robots that will perform their duties in an efficient manner”*
**Positive** **Affect**	Positive affect refers to the dimension that captures individuals’ emotional or affective responses characterized by positive feelings, attitudes, or sentiments toward the robot.	Inductive	Schaefer [68], Hancock et al. [69], Tay et al. [70], Wheless and Grotz [71], Colquitt and LePine [72], Yogoda and Gillan [73], Bhattacherjee [39], Büttner and Göritz [74]
		Deductive	*“Overall, the robot should be cute as that makes me feel protective of it and more trustful”* *“The robot looks like a robot it feels more honest and open”*
**Anthropomorphism**	Anthropomorphism refers to the extent that a robot has a human-like appearance, behavior, or personality in order to facilitate social interaction with humans.	Inductive	Ho and MacDorman [36], Walters et al. [75]
		Deductive	*“If it’s making a poor attempt at looking humanlike I immediately distrust it and am afraid of it”* *“Trusting robots that look like classic robots is easier than a robot that is made to look like a human”*

(4) Meaning Extracting.

In order to gain insight into the abstract opinions on facial anthropomorphic trustworthiness, we invited two experienced qualitative researchers to retrieve the relevant intuitions from the clusters. Together with the results from the pilot study and inductive approach, four themes were similarly identified and resulted in the generation of 82 items in total (see Table 1 and Table A1).

### 4.2. Phase 2: Item Refinement and Polish

Item refinement follows a two-step process. To specify, a group of five professors and Ph.D. candidates in various disciplines, such as design, sociology, and business, at a major university in Hong Kong evaluated the content validity of the items. Every researcher was informed with a written definition of facial anthropomorphic trustworthiness for social robots and four dimensions associated with an example item. Next, following the suggestions by Blijlevens et al. [76] and Bloch et al. [77], researchers were asked to classify each of the 82 items into one of four dimensions or a “none of these” category. Accordingly, items were removed when at least four out of five researchers could not reach a consensus on a particular category. Thus, this process resulted in 45 remaining items.

Then, the 45 items of four dimensions served as the input for the second step, a categorization task. Ten more researchers (different from the above researchers) with various disciplinary backgrounds were informed of the definition of each dimension and then asked to rate the extent of representation of the remaining items on a 5-point Likert scale (1 = “not representative of the dimension”, 5 = “very representative”). The average scores of each item served as the reference for the panel discussion among the ten researchers. The panel discussion aimed to negotiate and determine whether the item was representative enough of the given dimension until reaching a consensus [78]. This process resulted in 23 items. Then, two native English-speaking researchers modified the items to the given structure which served as the input for the item reduction (i.e., “this robot looks [adjective]”).

### 4.3. Phase 3: Item Reduction and Exploratory Factor Analysis

This phase consisted of item reduction and exploratory factor analysis. The process and stimuli were the same as in the pilot studies. Participants were informed that they would see and evaluate a set of social robot faces. Upon exposure to the stimuli, they were requested to specify their agreement with a set of items via a 9-point Likert scale (1 = strongly disagree, 9 = strongly agree). A total of 154 people were recruited from the crowdsourcing platform, AMT. Of these 154 participants, incomplete responses or responses with only consecutive or extreme values (1–9) were screened out from the questionnaire. The EFA analysis was conducted with a total of 125 participants (mean age = 36.35, SD = 10.29; 77 males and 48 females; education level: 12 with high school education or lower, 48 with some college, 65 with college education or above; robot use experience: 102 with no use, 16 with 0–1 year of use (1 year not included), 6 with 1–2 years of use (2 years not included), 1 with more than 2 years of use).

All items were scored on a 9-point Likert scale where higher scores suggested a higher level of facial anthropomorphic trustworthiness for a social robot. Before the exploratory factor analysis, we initially ran the Kaiser–Meyer–Olkin (KMO) measure of sampling adequacy and Bartlett’s test of sphericity to check whether the sample was appropriate for EFA [57]. The results showed that the KMO value was 0.914 and Bartlett’s test was significant (*p* < 0.001), suggesting that the current dataset was appropriate for EFA. Following the initial item reduction in the scale development procedure [77], the corrected item-total correlation estimation was calculated on the whole set of 23 items. Considering the threshold of the corrected item-total correlations (0.4 or above), two items were removed since they did not meet satisfactory item-total correlations. Then, a preliminary EFA via varimax rotation was conducted on the remaining set of 21 items. Two items were removed since they were conceptually irrelevant to the other items loaded on the specific construct: “typical representation” (construct typicality) and “diverse representation” (construct variety) [76]. One item was removed due to its failure to exhibit a simple structure on any factors [77]. Again, we re-performed the EFA on the remaining set of 17 items. All the corrected item-total correlations were beyond the threshold; thus, the remaining 17 items were retained. The EFA indicated a 17-item scale with four dimensions (cluster relied on its eigenvalues were one and above): ethics concern, capability, affective perception, and the uncanny valley effect. Regarding the Cronbach alpha values of each dimension, 0.89 was for “ethics concern”, 0.94 was for “capability”, 0.94 was for “positive affect”, and 0.88 was for “anthropomorphism” (Table 2).

### 4.4. Phase 4: Validation Phase

The same survey procedure was conducted in Phase 4: the same stimuli exposure, screening, and recruitment process was conducted to examine the final set of 17 items. As a result, 282 participants were included in the validation phase (mean age = 36.38, SD = 10.65; 177 male and 105 females; education level: 27 with high school education or lower, 81 with some college, 174 with college education or above; robot use experience: 201 with no use, 53 with 0–1 year of use (1 year not included), 19 with 1–2 years of use (2 years not included), 9 with more than 2 years of use).

Before the main analysis of confirmatory factor analysis (CFA), we ran EFA first to confirm the validity of four dimensions. Based on the eigenvalues (greater than one), the current result was consistent with the conclusion of Phase 3, suggesting the same four dimensions with the same items. The exploratory factor analysis of the final scale is given in Table 3. The results show that factor loadings for specific items contribute most strongly to each dimension, suggesting that the dimensions are conceptually distinct and do not have equal weights in assessing social robot trustworthiness.

As for CFA, the analysis was conducted through AMOS 25 for structural equation modeling (SEM) [79]. Particularly, SEM was utilized to examine whether the proposed model was structurally fitted with the sample. Specifically, the identified items on the same factors from EFA should be treated as the proposed model in the validation phase. Thus, the four-factor model (ethics concern, capability, positive affect, and anthropomorphism) from Phase 3 was used to test the data obtained in the second study through SEM [76].

**Figure 5:**
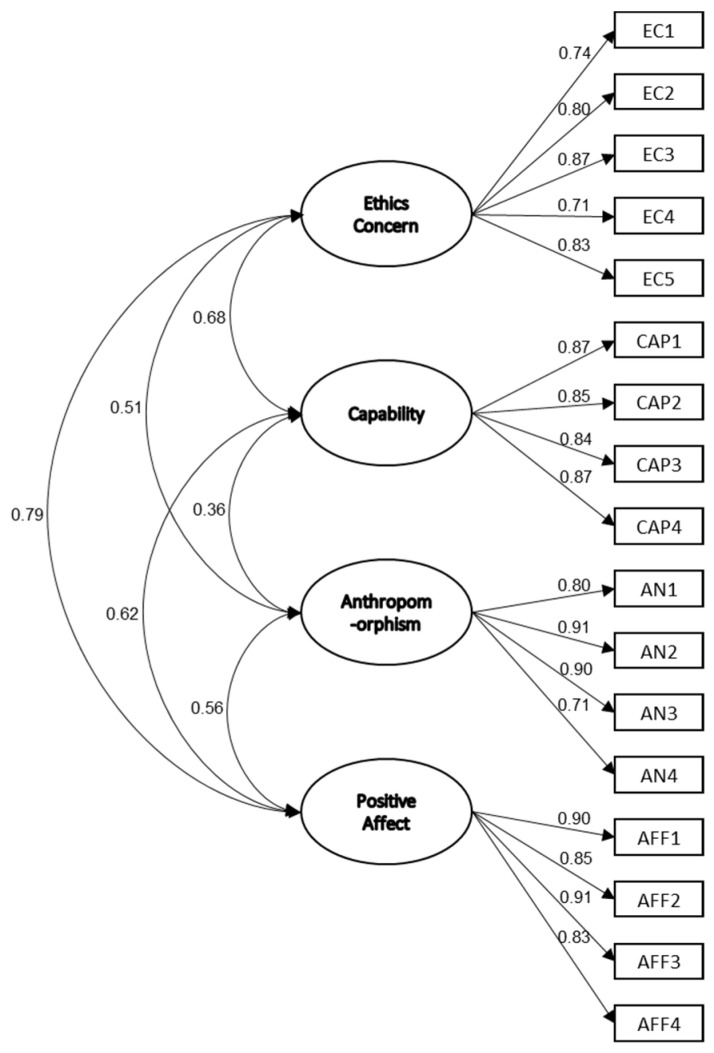
The path diagram of facial anthropomorphic trustworthiness scale.

According to the result, it was shown the model fit index achieved adequate values, confirming the general appropriateness of the model from EFA [80]. To specify, the goodness-of-fit measure (GFI) was 0.892 (threshold: 0.9 and above), the incremental fit index (IFI) was 0.957 (threshold: 0.9 and above), the normed fit index (NFI) was 0.929 (threshold: 0.9 and above), the comparative fit index (CFI) was 0.957 (threshold: 0.9 and above), the adjusted goodness-of-fit measure (AGFI) was 0.854 (threshold: 0.8 and above), and the root-mean-square error of approximation (RMSEA) was 0.07 (threshold: 0.05–0.08). In addition, all items had significant factor loadings, varying from 0.71 to 0.91 (Figure 5), and all explained variances (squared multiple correlations, SMC) of our items varied between 0.49 and 0.83.

As for reliability and convergent validity, the average variance extracted (AVE) for every construct had achieved a satisfactory value (0.50 and above), suggesting that the current sample had sufficient convergent validity [80]. As suggested by Fornell and Larcker [80], the composite reliability (C.R.) of each construct achieved an adequate value (0.60 and above).

Regarding the discriminant validity within the model, the discriminant validity between the four constructs was measured by whether the square root of the AVE (diagonal values in Table 4) was larger than the rest of the inter-construct correlations and the maximum shared variance (MSV) [80]. The results suggested that all four constructs reached adequate discriminant validity, suggesting that the dimensions are not equally related.

## 5. Discussion

With the increasing application of technology and equipment in social robots, they are becoming communication partners and mediums between humans and digital data in our daily lives. These robots provide support in both physical and emotional ways [81]. Considering the significant role of ethics evaluation in the initial stages of human–robot interaction (HRI), facial anthropomorphic trustworthiness plays a crucial role in establishing initial credibility and fostering approaching intentions in later stages [81,82] (page 177 and page 7, respectively). However, within the domain of social robots, there is no valid scale to measure facial anthropomorphic trustworthiness. To address this research gap, this study developed a reliable and valid measurement for assessing facial anthropomorphic trustworthiness in the context of social robots. The resulting scale can be utilized in future empirical studies on HRI to assess the determinants that influence trustworthiness, particularly at first sight.

The study identified four dimensions that can be applied to assess facial anthropomorphic trustworthiness: ethics concern, capability, positive affect, and anthropomorphism. These dimensions align with the Theory of Robot Communication (TORC), which suggests that humans interact with social robots through two parallel paths: robot-mediated communication (a slow and reflective route) and human–robot communication (a quick and effective route) [81,82] (page 178 and page 7, respectively). Individuals may simultaneously engage in both routes, with one dominating or switching with the other. These findings are consistent with the dimensions identified in the current study: the affective route (ethics concern and positive affect) and the reflective route (capability and anthropomorphism).

This study contributes to the literature on HRI and scale development in several ways. Firstly, it introduces a facial anthropomorphic trustworthiness scale that integrates theories of interpersonal trustworthiness, the uncanny valley effect, and general robot trustworthiness. By considering these theoretical perspectives, our scale provides a more comprehensive perspective when evaluating the trustworthiness of a social robot based on first impressions. This approach distinguishes our study by incorporating multiple dimensions of trustworthiness, which may not have been explored in previous works.

Secondly, the study employed a relatively large sample of qualitative data for the item-generation process. This approach allowed for a deeper understanding of the nature of facial anthropomorphic trustworthiness. While traditional qualitative methods can be costly and time-consuming, we leveraged innovative techniques such as natural language processing to analyze the data in a more objective manner [83]. This utilization of advanced computational methods for quantifying qualitative data represents an emerging field that enables researchers to gain insights from large-scale qualitative datasets.

Moreover, in the context of AI-based systems, it is essential to consider the advantages and restrictions of our proposed method. The incorporation of AI and machine calibration technologies in social robots introduces additional considerations for trustworthiness and explainability. Understanding the limitations and opportunities of AI-based systems can help refine the proposed scale. We can draw insights from the literature, such as the challenges and opportunities outlined in the field of AI and machine learning [84].

Furthermore, considering the growing importance of Explainable Artificial Intelligence (XAI) in enhancing transparency and accountability in AI systems, it is relevant to discuss how our proposed facial anthropomorphic trustworthiness scale aligns with the goals of XAI. By incorporating concepts and ideas from the systematic review of XAI models and applications [85], we can highlight how our scale contributes to the explainability of social robots’ behavior and decision-making processes. This aligns with the broader goals of XAI in promoting trust and understanding in AI-based systems.

However, there are also some points worth noting. Though the current study used a relatively large sample of qualitative data to gain a deep understanding of the nature of facial anthropomorphic trustworthiness, it would be interesting to apply this innovative approach to explore other significant issues, such as the implicit dimensions for robot preference, which could, in turn, strengthen our understanding of trustworthiness in human–robot interactions.

## 6. Conclusions

In conclusion, social robots have emerged as autonomous systems capable of performing social behaviors and assuming social roles. However, despite their potential to revolutionize human–robot interactions, there is a paucity of research focusing on the specific measurement of facial trustworthiness toward anthropomorphic robots, particularly during initial interactions. To bridge this research gap, a hybrid approach was employed in this study, utilizing a crowdsourcing platform for data collection and deep convolution and factor analysis for data processing. The aim was to develop a comprehensive scale, named Facial Anthropomorphic Trustworthiness towards Social Robots (FATSR-17), to accurately measure the trustworthiness of a robot’s facial appearance. The final measurement scale consists of four dimensions, namely, ethics concern, capability, positive affect, and anthropomorphism, and comprises 17 items. An iterative examination and refinement process was conducted to ensure the reliability and validity of the scale. This study contributes to the field of robot design by providing designers with a structured toolkit to create social robots that appear trustworthy to users, thus enhancing the potential for successful human–robot interactions.

## Figures and Tables

**Table 2:** The 17 items of the final scale.

**Ethics Concern (5)**	**Capability (4)**
EC1. This robot does not look evil	CAP1. This robot looks competent in its work
EC2. This robot looks as if its creator is not intending to harm humanity	CAP2. This robot looks like it can perform its duties in an efficient manner
EC3. The designer has ethically programmed this robot	CAP3. This robot looks like it can be successful in the matter it is programmed to do
EC4. This robot seems to act following its program	CAP4. This robot looks like it can provide appropriate information
EC5. This robot seems reasonable when interacting with a human	
**Positive Affect (4)**	**Anthropomorphism (4)**
AFF1. This robot looks kind	AN1. This robot face looks neither too living nor too inanimate
AFF2. This robot looks cute	AN2. This robot face looks neither too humanoid nor too robotic
AFF3. This robot looks considerate	AN3. This robot face looks neither too real nor too synthetic
AFF4. This robot looks like it cares about my welfare	AN4. This robot face strikes a balance between a human-like face and a machine-like face

**Table 3:** The exploratory factor analysis of the scale.

	Factors			
	Capability	Ethics Concern	Anthropomorphism	Positive Affect
**CAP1**	0.848			
**CAP2**	0.794			
**CAP3**	0.766			
**CAP4**	0.752			
**EC2**		0.752		
**EC1**		0.696		
**EC5**		0.653		
**EC3**		0.635		
**EC4**		0.615		
**AN2**			0.898	
**AN3**			0.832	
**AN1**			0.725	
**AN4**			0.673	
**AFF3**				0.744
**AFF2**				0.743
**AFF1**				0.715
**AFF4**				0.692

Note: Extraction Method: Principal axis factoring. Rotation Method: Varimax with Kaiser normalization. Rotation converged in six iterations with loading values of more than 0.5.

**Table 4:** Reliability and validity test of constructs.

	CR	AVE	MSV	EC	CAP	AN	AFF
**Ethics Concern (EC)**	0.89	0.63	0.63	0.79			
**Capability (CAP)**	0.92	0.74	0.46	0.68 ***	0.86		
**Anthropomorphism (AN)**	0.90	0.69	0.31	0.51 ***	0.36 ***	0.83	
**Positive Affect (AFF)**	0.93	0.76	0.63	0.79 ***	0.62 ***	0.56 ***	0.87

Note: *** *p* < 0.01.

## Data Availability

Data will be provided upon request.

## List of Abbreviations

**Abbreviations**	**Definition**
A.I.	Artificial Intelligence
FATSR-17	Facial Anthropomorphic Trustworthiness towards Social Robots
HRI	Human–robot interaction
EFA	Exploratory factor analysis
CFA	Confirmatory factor analysis
NLP	Natural language processing
SOTA	State-of-the-art
UGC	User-generated content
BERT	Bidirectional encoder representations from transformers
MLM	Mask language modeling
NSP	Next sentence prediction
EC	Ethics concern
CAP	Capability
AFF	Positive affect
AN	Anthropomorphism
SEM	Structural equation model
GFI	Goodness of fit
IFI	Incremental fit index
NFI	Normed fit index
CFI	Comparative fit index
AGFI	Adjusted goodness of fit measure
RMSEA	Root-mean-square error of approximation
AVE	Average variance extracted
MSV	Maximum shared variance
TORC	Theory of robot communication

## Appendix A

**Table A1:** Original Item Pool.

Ethics Concern	Capability	Positive Affect	Anthropomorphism
look evil	capable	friendly	uncanny
sufficient integrity	competent	conscious	an actual human face
self-awareness	perform duties	kind	natural appearance
ethics in programing	successful at the things	cute	appropriate features
sense of justice	sufficient artificial intelligence	happy	too human-identical
stick to its program	confident	well qualified	too close to humanity
looks fair	specialized capability	concerned welfare	a minimum of human appearance
behaviors are consistent	satisfy users’ needs	desires seem important	machine-like features
ethical concern	expect good advice	anything to hurt me	moderate lifelike
sound principles	dependable	important to me	weird face
predictable	reliable	help me	complex detailed features
standards	autonomous	interested in welfare	neither too plain nor too weird
operates scrupulously	finish the task	put my interest first	neither too dull nor too freaky
statements	follow the advice	responsible	too boring nor too shocking
methods are clear	give me advice	supportive	balance between human and machine
keeps promises	rely on the advice	pleasant	neither too real nor too synthetic
protect human	function successfully	join our team	infantile like
openly communicate	clearly communicate	aggressive	neither too humanoid nor too robotic
perform as instructed	frequent maintenance		neither too living nor too inanimate
obey order	better than a novice human user		
a competitor for job	provide feedback		
hacked easily	meet the need of the mission		
	provide appropriate information		
